# The *dnd* operon for DNA phosphorothioation modification system in *Escherichia coli* is located in diverse genomic islands

**DOI:** 10.1186/s12864-015-1421-8

**Published:** 2015-03-17

**Authors:** Wing Sze Ho, Hong-Yu Ou, Chew Chieng Yeo, Kwai Lin Thong

**Affiliations:** Institute of Biological Sciences, Faculty of Science, University of Malaya, 50603 Kuala Lumpur, Malaysia; State Key Laboratory for Microbial Metabolism and School of Life Sciences & Biotechnology, Shanghai Jiaotong University, 200030 Shanghai, China; Biomedical Research Centre, Faculty of Medicine, Universiti Sultan Zainal Abidin, 20400 Kuala Terengganu, Malaysia

**Keywords:** DNA degradation, Dnd, *Escherichia coli*, PFGE, Thiourea, Genetic environment, Genomic islands, *Dnd*-encoding genomic island

## Abstract

**Background:**

Strains of *Escherichia coli* that are non-typeable by pulsed-field gel electrophoresis (PFGE) due to in-gel degradation can influence their molecular epidemiological data. The DNA degradation phenotype (Dnd^+^) is mediated by the *dnd* operon that encode enzymes catalyzing the phosphorothioation of DNA, rendering the modified DNA susceptible to oxidative cleavage during a PFGE run. In this study, a PCR assay was developed to detect the presence of the *dnd* operon in Dnd^+^*E. coli* strains and to improve their typeability. Investigations into the genetic environments of the *dnd* operon in various *E. coli* strains led to the discovery that the *dnd* operon is harboured in various diverse genomic islands.

**Results:**

The *dndBCDE* genes (*dnd* operon) were detected in all Dnd^+^*E. coli* strains by PCR. The addition of thiourea improved the typeability of Dnd^+^*E. coli* strains to 100% using PFGE and the Dnd^+^ phenotype can be observed in both clonal and genetically diverse *E. coli* strains.

Genomic analysis of 101 *dnd* operons from genome sequences of *Enterobacteriaceae* revealed that the *dnd* operons of the same bacterial species were generally clustered together in the phylogenetic tree. Further analysis of *dnd* operons of 52 *E. coli* genomes together with their respective immediate genetic environments revealed a total of 7 types of genetic organizations, all of which were found to be associated with genomic islands designated *dnd*-encoding GIs. The *dnd*-encoding GIs displayed mosaic structure and the genomic context of the 7 islands (with 1 representative genome from each type of genetic organization) were also highly variable, suggesting multiple recombination events. This is also the first report where two *dnd* operons were found within a strain although the biological implication is unknown. Surprisingly, *dnd* operons were frequently found in pathogenic *E. coli* although their link with virulence has not been explored.

**Conclusion:**

Genomic islands likely play an important role in facilitating the horizontal gene transfer of the *dnd* operons in *E. coli* with 7 different types of islands discovered so far.

**Electronic supplementary material:**

The online version of this article (doi:10.1186/s12864-015-1421-8) contains supplementary material, which is available to authorized users.

## Background

Microbial source tracking (MST) is important to trace the source of a pathogen in an outbreak situation. Pulsed field gel electrophoresis (PFGE) is one of the tools for MST and is the method of choice for a number of bacterial pathogens as it is highly discriminative, reproducible [1] and standardized protocols are available. However, certain bacterial species such as *Escherichia coli*, *Pseudomonas aeruginosa*, *Klebsiella pneumoniae* and many more are susceptible to DNA degradation, resulting in smeared DNA, which can be improved by the addition of thiourea into the PFGE running buffer [2-4]. The *DN*A *d*egradation phenotype (designated Dnd) was first observed during an electrophoretic separation of DNA of a Gram-positive bacterium, *Streptomyces lividans* [5]. A DNA modification system (conferred by a five-gene *dnd* cluster, *dndABCDE*) was then found to be responsible for the Dnd phenotype by mediating the incorporation of sulphur into the DNA backbone via a process called phosphorothioation [6,7]. The DNA modification renders the DNA susceptible to cleavage by peracetic acid generated during a PFGE run [8,9]. While the 5-gene cluster (consisting of *dndA* and the *dndBCDE* operon) is found in bacteria across different families (such as *Acidobacteriaceae*, *Clostridiaceae*, and *Streptomycetaceae*), *Enterobacteriaceae* including *E. coli* only harbour a 4-gene *dndBCDE* operon, lacking the *dndA* gene. IscS, a cysteine desulfurase, which has a similar function as DndA was then identified in *E. coli* to support DNA phosphorothioation [10,11].

*dnd* operons are often located in chromosomal islands, where highly diverse genetic contexts were observed across different bacterial species [10]. However, there is dearth of studies on the diversity of the *dnd*-encoding GIs from the same bacterial species. To date, only one *dnd*-encoding genomic island has been described for the enteric pathogen *Salmonella enterica* [10]. A more distantly related bacterium, *Mycobacterium abscessus*, was reported to have conserved regions flanking their *dnd*-encoding GIs but the description of the *dnd*-encoding GIs itself was not available [12]. The lack of data regarding the genetic environment of *dnd* operons makes comprehensive comparative analysis between the same and different bacterial species difficult. Although there are ample bacterial genome sequences in the public domain, comparative studies on the *dnd* clusters of *E. coli* are still lacking. In this study, we aimed to (1) improve the typeability of *E. coli* affected by DNA degradation (Dnd^+^); (2) investigate the genetic diversity of Dnd^+^*E. coli* strains using PFGE; (3) develop a PCR assay to detect the *dnd* operons in Dnd^+^*E. coli*; (4) determine the association between the presence of the *dnd* operon and the Dnd phenotype; (5) determine the genetic diversity and genetic environment of *dnd* operons; (6) investigate the background of *E. coli* harbouring *dnd* operons. The outcome of the study may provide further insights into the diversification of *dnd* operons in *E. coli*.

## Results

### Addition of thiourea improved the typeability of Dnd^+^ strains by 100%

**Figure 1:**
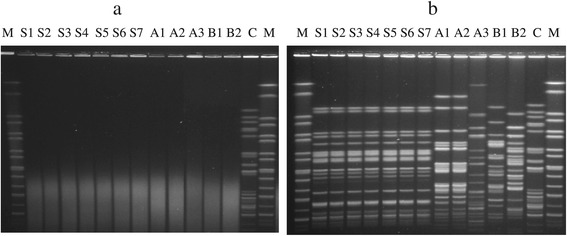
**Effects of thiourea improved the typeability of Dnd** ^**+**^ **strains by PFGE. a:** PFGE gel obtained by performing PFGE run without the addition of thiourea. **b:** PFGE gel obtained by performing PFGE run with the addition of thiourea to the agarose gel and running buffer. Lane M: *XbaI* digested H9812 DNA marker; lanes S1-S7, VTEC isolates from pigs; lanes A1-A3, clinical isolates obtained from medical center A; lanes B1-B2, clinical isolates obtained from medical center B; C, control *E. coli* isolate which is typeable with and without the addition of thiourea (**a** and **b**). Gel images were captured using GelDoc (BioRad, Hercules, CA) digital gel documentation system.

Without the addition of thiourea to the PFGE run, all 12 Dnd^+^*E. coli* strains from previous studies [13,14] yielded degraded DNA despite repeated attempts (3 times) (Figure 1(a), lanes S1-B2). However, when thiourea was added to the running buffer, there was a 100% marked improvement in typeability for all the *E. coli* strains (Figure 1(b), lanes S1-B2). Strains with *Bln*I-digested chromosomal DNA were also typeable when thiourea was added in the PFGE run (data not shown). Seven zoonotic VTEC strains (lanes S1-S7) and two clinical strains (lanes A1-A2) shared two indistinguishable pulsotypes (i.e., clonal) (Figure 1(b)) while another 3 (lanes A3, B1, B2) were genetically different.

### Sequence analysis of *dndB*-*E* genes and association of the DNA degradation phenotypes and genotypes

PCR detection of *dndC* gene using primers described previously [15] revealed that out of 12 Dnd^+^*E. coli* strains, only 7 zoonotic VTEC (Figure 1, lanes S1-S7) and 2 clinical (Figure 1, lanes B1-B2) Dnd^+^ strains were positive for the *dndC* gene. Another 3 Dnd^+^ strains (A1-A3) were negative for the *dndC* gene and these strains were obtained from the same medical center and source. The failure to amplify the *dndC* gene from 3 out 12 Dnd^+^ strains using previously described primers [15] prompted us to develop a PCR assay targeting the internal regions of *dndB*, *dndC*, *dndD*, and *dndE* based on the *dndBCDE* DNA sequences of *E. coli* available in dndDB [10]. All 12 Dnd^+^ strains yielded the expected PCR amplicons for all 4 genes (*dndB*, *dndC*, *dndD*, and *dndE*) (using the designed primers listed in Table 1). All Dnd^−^ strains (including *E. coli*, *Shigella sonnei*, *Salmonella* Typhimurium and *Salmonella* Enteriditis) that did not display degradation phenotype were negative for the *dnd* genes. Significant correlation was also found between the presence of the *dnd* gene cluster and the Dnd phenotype (p < 0.05).

**Table 1:** **Primers used for detection of** ***iscS*** **genes and genes in** ***dnd*** **operon**

**Primer**	**Oligonucleotide sequence (5' to 3')**	**Target gene**	**Amplicon size (bp)**	**Annealing temperature (°C)**	**Reference(s)**
**IscS-CMu F**	ATCTGACAACCTGGCGATCA	*iscS*	860	44	An *et al*., 2012 [11]
**IscS-CMu R**	CTTCAGTAGTAAAACGACCT				
**dndC-F**	CCGTATCGGTCGCATTGTT	*dndC*	579	51	Wang *et al*, 2011 [15]
**dndC-R**	CTTCGTCCCATTTCAGGTT				
**dndB-F**	CATTGCCAGATAACGCGCC	*dndB*	207	50	This study
**dndB-R**	CGTAAAGCGATTGAAGACGC				
**dndC-F**	TTTCCACCCCACGGGCTTTC	*dndC*	532	56	This study
**dndC-R**	GCCTGCCCCCGGAAATG				
**dndD-F**	AGCCTCCTT(A/T)GTTAATTCCCAGA	*dndD*	605	51	This study
**dndD-R**	AGCAACTGGA(T/C)TGTGT(C/T)CGT				
**dndE-F**	GC(A/G)AT(C/T)CCATCCTC(C/A)ACAT	*dndE*	196	48	This study
**dndE-R**	GCTCGGTGGAGAGTGAGT				

At least two sets of the amplified *dndB*-*dndE* genes from different strains with a similar pulsotype were sequenced. It was found that the *dndB*-*dndE* genes from different strains with the same pulsotype shared identical (100%) nucleotide sequences. Multiple sequence alignment (MSA) of the nucleotide sequences of the *dndB*-*dndE* genes with *E. coli* SE11, 55989 and B7A were also carried out. Using *E. coli* 55989 as reference, the pairwise alignments showed that *E. coli* S1-S7, B1 and B2 shared *dndB*-*E* sequences that are highly similar to *E. coli* 55989 (BLASTN identities of *dndB*-*C* = 100%; *dndD* = 99.8% and *dndE* = 98.5%). Strains A1 and A2 harboured identical *dnd* genes but are different from *E. coli* 55989 (*dndB* = 89.9%; *dndC* = 85.2%; *dndD* = 98.2; *dndE* = 95.9%). The *dndB*-*E* sequences of strain A3 are most distantly related to those of *E. coli* 55989 (*dndB* = 87.9%; *dndC* = 89.6%; *dndD* = 80.6%; *dndE* = 84%) but are highly similar to *E. coli* B7A (identical *dndB*-*D* sequences with 2 SNPs with respect to *dndE*) (Additional file 1: Figure S1). Hence, the inability to amplify the *dndC* genes using primers described previously [15] for strain A1-A3 might be due to the diversity in the sequences of the *dnd* operons in various *E. coli* strains. Further *in silico* analysis also revealed that mismatches at the primer priming sites (n = 2 – 10) were found for *E. coli* 55989, SE11 and B7A. However, it should be noted that primers described by Wang et al., (2011) [15] were for the expression studies of the *dnd* operon from *Salmonella enterica* (although closely related but not identical to *E. coli*). Furthermore, to the best of our knowledge, there are no other appropriate primers available for the detection of the *dndBCDE* gene cluster in *E. coli*. It should also be noted that the DNA sequences of the *dndB*-*E* genes included in the MSA are partial CDS. The DNA sequences of *dndB*-*dndE* genes from each pulsotype have been deposited in GenBank under accession nos. KJ702391-KJ702410.

PCR detection of *iscS* revealed that all *E. coli* (both Dnd^+^ and Dnd^−^ strains), *Salmonella* spp. and *Shigella* strains were positive for the *iscS* gene. In fact, *iscS* is also found in *Citrobacter* spp., *Klesiella* spp., and *Morganella* spp. using BLASTN indicating that this cysteine desulfurase gene may be ubiquitous in different enterobacterial strains, thus supporting its suggested importance in sustaining fundamental life processes [16]. However, this also indicates that the presence of the *iscS* gene is not a good genotypic marker for the Dnd^+^ phenotype. Representative *iscS* sequence obtained from this study have been deposited in GenBank under accession no KC839813.

### *dnd* operons from different bacterial genera were grouped into separate distinct clusters

A total of 106 positive hits (with BLASTN e-value = 0, identity ≥ 85% and coverage ≥98%) were obtained from both databases of non-redundant nucleotide collection (nr/nt) and whole genome shotgun contigs (WGS) using the three *dnd* operons (*E. coli* SE11, 55989 and B7A) as queries. All three queries also yielded the same number of positive hits including genomes from other species from *Enterobacteriaceae* besides *E. coli*, including *Serratia* spp., *Erwinia* spp. and *Citrobacter* spp.. Only 101 out of 106 *dnd* operons were included in the comparative analysis because the remaining *dnd* operons were located in the gaps of their respective draft genomes and were thus, incomplete. The accession numbers of all 106 genomes with positive hits were summarized in Additional file 2: Table S1 which also included the *E. coli* strain background information.

**Figure 2:**
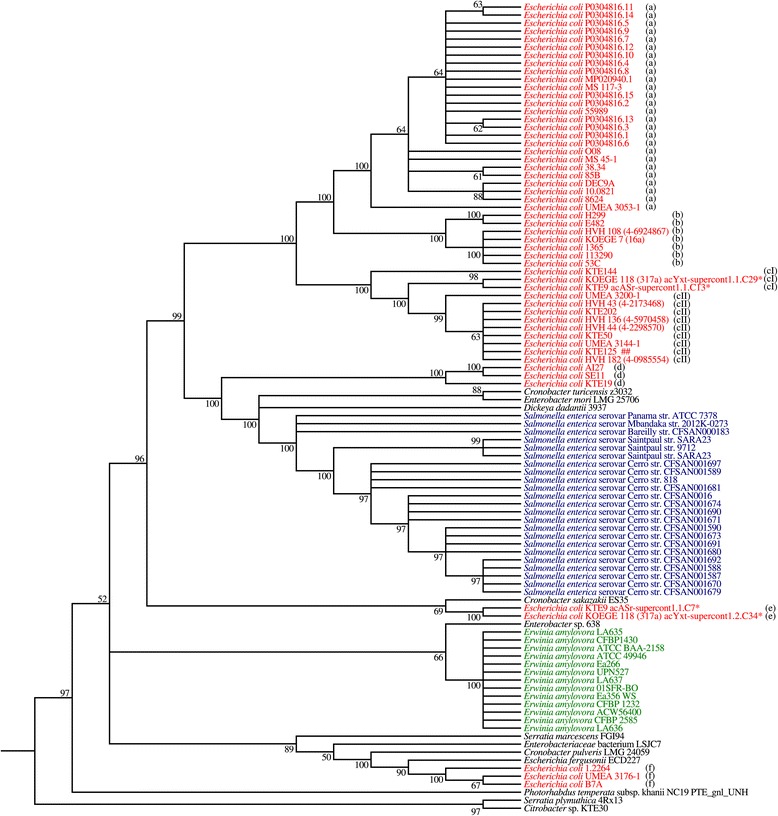
**Phylogenetic tree of** ***dnd*** **operons from** ***Enterobacteriaceae*** **.** Asterisk (*) indicates that the genome contains two copies of *dnd* operons. Different alphabets **(a, b, cI, cII, d, e, f)** depict the subgroups based on the immediate genetic environment. Colored genome strain codes facilitate visualization. Maximum likelihood (ML) method was used to construct the phylogenetic tree using MEGA5. Bootstrap confidence values greater than 50% are shown at branches. Nodes with less than 50% bootstrap value are collapsed.

A phylogenetic tree was constructed using the multiple sequence alignment of the nucleotide sequences of the 101 *dnd* operons (Figure 2). All *dnd* sequences from *Salmonella* spp. (blue) and *Erwinia amylovora* (green) were grouped into two distinct separate clusters. On the other hand, the *dnd* sequences from the genomes of other *Enterobacteriaceae* such as *Citrobacter* spp., *Cronobacter* spp., *Enterobacter* spp. and *Serratia* spp. were generally not clustered into any groups (Figure 2). As for *E. coli* (red), 45 out of 53 of them were grouped together into one main cluster. The remaining 8 were placed at different nodes. Another phylogenetic tree was constructed using the nucleotide sequences of 100 *dnd* operons (*E. coli* MS45-1 was excluded because the immediate vicinity of its *dnd* operon were located in the gaps, hence the total number of *E. coli* genomes in the analysis was reduced to 52) together with their respective immediate vicinity (1 kb) (Additional file 3: Figure S2) to determine whether the immediate genetic environment will cause any changes to the phylogenetic tree. Surprisingly, both trees (Figure 2 and Additional file 3: Figure S2) yielded highly similar results with only slight differences. For example, *Cronobacter turicensis* z3032 is more closely related to *Enterobacter mori* LMG 25706 (both forms a sister group) than to *Dickeya dadantii* 3937 by comparing their *dnd* operons only (Figure 2). On the other hand, *Cronobacter turicensis* z3032 is more closely related to *Salmonella enterica* strains when the *dnd* operons and their respective immediate vicinity were included in the analysis (Additional file 3: Figure S2). Overall, both trees yielded similar results.

Based on the sequence similarity of the immediate upstream and downstream regions (1 kb) of the 52 *dnd* operons of *E. coli*, a total of 7 subgroups were further identified. The 7 subgroups were designated a, b, cI, cII, d, e and f and were included in Figure 2. It was observed that genomes harbouring highly conserved *dnd* operons also shared similar immediate genetic environments and vice versa. Of the 7 subgroups identified, groups cI and cII share similar immediate downstream but different upstream regions while all other groups were very different with respect to their immediate genetic environment. Interestingly, 4 of the 52 *dnd* operons from *E. coli* genomes belonged to two *E. coli* draft genomes (KOEGE 118 and KTE 9). MSA analysis revealed that the two copies of the *dnd* operon and their associated genetic environment in the same *E. coli* genome are different and are distantly located from each other in the phylogenetic tree generated (groups cI and e) (Figure 2 and Additional file 3: Figure S2).

### Upstream and downstream regions of 12 Dnd^+^*E. coli* strains resemble three different types of genetic environment

PCR detection of the immediate upstream and downstream regions of the *dnd* operon for the 12 Dnd^+^*E. coli* strains revealed three types of immediate genetic environment. Strains S1-S7, B1 and B2 shared *dnd* operons with associated genetic environment similar to group a; A1 and A2 to cII; and A3 to group f. All three types of immediate genetic environment identified in the Dnd^+^*E. coli* strains using PCR matched to those determined by *in silico* analysis, indicating that there were only 7 types of different immediate genetic environment identified to date for *E. coli*.

### Diverse *dnd*-encoding genomic islands were observed for various representative *E. coli* genomes

**Figure 3:**
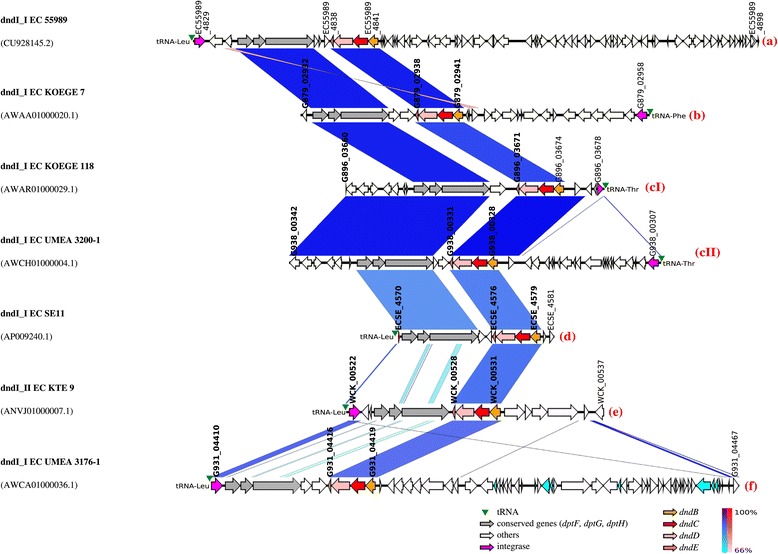
**Genetic map of 7 different** ***dnd-*** **encoding genomic islands of** ***E*** **.** ***coli*** **.** Same-strand DNA similarity is shaded blue (dark blue-sky blue) while reverse similarity is shaded red (red-light pink). Coding sequences are displayed as arrows. Major features are displayed and are colored to facilitate visualization. Order of genomic islands in the map was arranged according to the phylogenetic tree constructed using the *dnd* operons. Red alphabet in brackets indicates the subgroup determined based on the immediate genetic environment of the respective *dnd* operons. Integrase gene is the first element of the *dnd*-encoding genomic island (GI).

**Figure 4:**
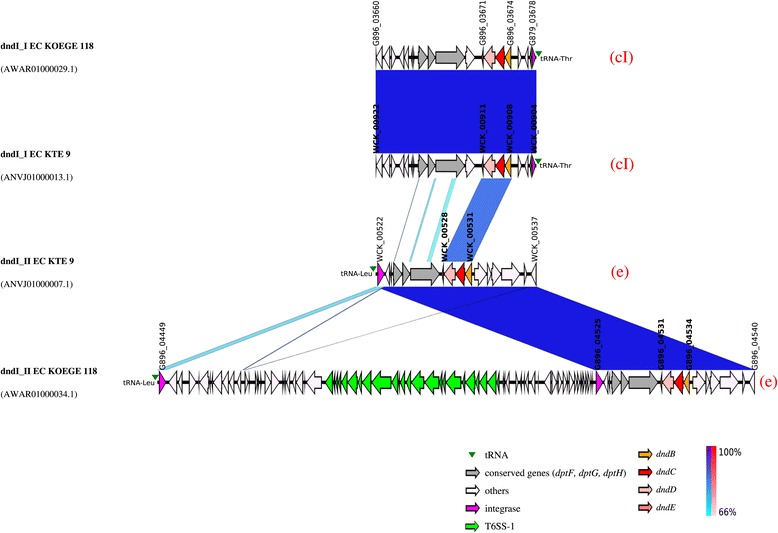
**Genetic map of the** ***dnd-*** **encoding genomic islands of** ***E*** **.** ***coli*** **KTE9 and KOEGE118 which harboured 2 copies of** ***dnd*** **operons.** The integrase gene is the first element of the *dnd*-encoding genomic island (GI) although the genetic map is illustrated according to the orientation of the *dnd* operons to facilitate visualization. Same-strand DNA similarity is shaded blue (dark blue-sky blue) while reverse similarity is shaded red (red-light pink). Coding sequences are displayed as arrows. Major features are displayed and are colored to facilitate visualization. Red alphabet in brackets indicates the subgroup determined based on the immediate genetic environment of the respective *dnd* operons. An additional DNA segment of 74 kb was observed for dndI_II_EC KOEGE 118_ compared to dndI_II_EC KTE 9_. dndI_I_EC KOEGE 118_ is identical to dndI_I_EC KTE 9_.

**Figure 5:**
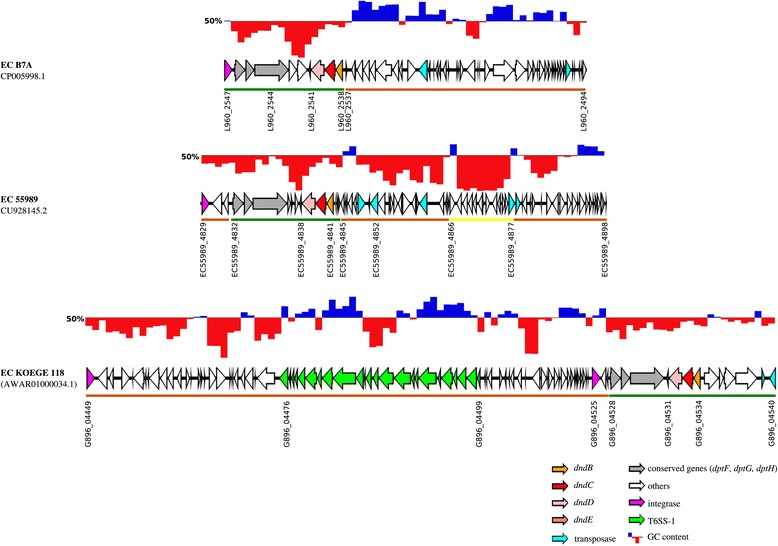
**GC content of three representative** ***dnd*** **-encoding genomic islands.** GC content of *dnd*-encoding GIs are shown above their respective genetic maps, with blue and red region showing GC content above and below 50%, respectively. The *dnd*-encoding GIs consist of several modules, depicted by the coloured bars below their respective genetic maps. ORFs above the brown coloured bars were found in both *dnd*-positive and *dnd*-negative *E. coli* genomes. ORFs that are underlined by green bars are strictly associated with *dnd*-positive genomes only while ORFs underlined by the yellow bar are strain-specific.

**Figure 6:**
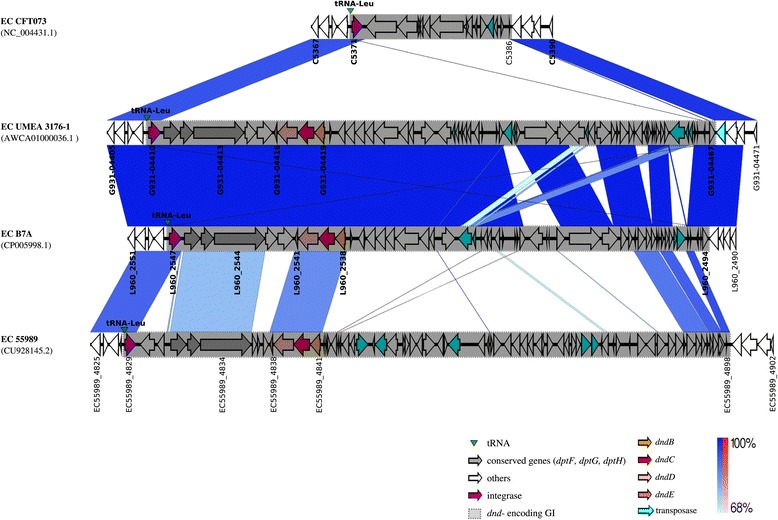
**Example of different genetic contents at the** ***leu*** **tRNA insertion sites of** ***dnd*** **-negative and** ***dnd*** **-positive** ***E*** **.** ***coli*** **genomes.** Same-strand DNA similarity is shaded blue (dark blue-sky blue) while reverse similarity is shaded red (red-light pink). Coding sequences are displayed as arrows. Major features are displayed and are coloured to facilitate visualization.

A genome from each of the seven subgroups determined by the sequence similarity of their respective immediate genetic environment was selected for further genomic analysis. Only 1 genome was selected from each subgroup because it was impossible to determine all complete GIs carrying the *dnd* operon as most of the genomes in the public database were partially sequenced. All 7 *dnd* operons from the selected *E. coli* genomes were found to be associated with GIs. The 7 *dnd*-encoding GIs were then designated based on the naming system described in the Methods section. Thus, the *dnd*-encoding GI located in *E. coli* 55989 (CU928145.2) was designated “dndI_I_EC 55989_”; KOEGE 7 (16a) acYxM- supercont1.4.C20 (AWAA01000020.1) as “dndI_I_EC KOEGE 7_”; KOEGE 118 (317a) acYxt-supercont1.1.C29 (AWAR01000029.1) as “dndI_I_EC KOEGE 118_”; UMEA 3200-1 acYwY- supercont1.1.C4 (AWCH01000004.1) as “dndI_I_EC UMEA 3200-1_”; SE11 (AP009240.1) as “dndI_I_EC SE11_”, KTE9 acASr-supercont1.1.C7 (ANVJ01000007.1) as “dndI_II_EC KTE 9_” and UMEA 3176-1 acYyH-supercont1.8.C36 (AWCA01000036.1) as “dndI_I_EC UMEA 3176-1_”. The genetic maps of the 7 types of *dnd*-encoding GIs are illustrated in Figure 3. Based on the genetic maps of the 7 *dnd*-encoding GIs, it was found that the genetic contents of the GIs harbouring the *dnd* operons were variable (Figure 3) although all the GIs described were obtained from *E. coli*. This indicates the potential contribution of GIs to the evolution and diversification of closely related bacteria. All 7 *dnd*-encoding GIs were also associated with tRNA and integrase genes (Figure 3). Further exploration of the genetic environment revealed the presence of three conserved genes (*dptF*, *dptG*, *dptH*), found upstream of the *dnd* operons, where both gene clusters were often separated by 1 to 3 hypothetical proteins (Figures 3, 4, 5 and 6). The three conserved genes were found in the vicinity of the *dnd* operon of all *E. coli* genomes included in the MSA analysis using BLASTN with the exception of strains *E. coli* C496_10 and *E. coli* O08 where only draft genomes were available. Thus, we could not be certain if these genes were absent or they were lost in the gaps of the incomplete genome sequences.

*E. coli* KOEGE 118 and KTE 9 harboured 2 copies of *dnd* operons each, and all were associated with GIs as well. The *dnd*-encoding GIs in KOEGE 118 were designated dndI_I_EC KOEGE 118_ (acYxt-supercont1.1.C29; accession no, AWAR01000029.1) and dndI_II_EC KOEGE 118_ (acYxt-supercont1.2.C34; AWAR01000034.1) whereas in KTE9, they were designated dndI_I_EC KTE 9_ (acASr-supercont1.1.C13; ANVJ01000013.1) and dndI_II_EC KTE 9_ (acASr-supercont1.1.C7; ANVJ01000007.1) with their genetic maps depicted in Figure 4. Interestingly, both dndI_I_EC KOEGE 118_ and dndI_I_EC KTE9_ are identical whereas dndI-II_EC KTE9_ is identical to dndI-II_EC KOEGE 118_ (covering the *dnd* operon and its immediate vicinity) but dndI-II_EC KOEGE 118_ had an additional 74 kb segment containing a type VI secretion system (T6SS) that was absent from dndI-II_EC KTE9_ (Figure 4). Thus, in terms of the *dnd* operon and its immediate genetic vicinity, both dndI_II_EC KOEGE 118_ and dndI_II_EC KTE 9_ were grouped within the same group e (Figure 2 and Additional file 3: Figure S2). It should be noted that not all genomes from the same subgroup shared identical GIs as they are often very large in size, and variation may have occurred within the GIs caused by other mobile genetic elements [17].

### *dnd*-encoding genomic islands displayed mosaic structures

Due to the great diversity of the islands harbouring *dnd* operons, three representative islands from *E. coli* strains B7A, 55989 and KOEGE 118 belonging to different groups (Figure 2) were selected for further investigation to determine the evolutionary forces driving the great variation between the *dnd*-encoding GIs. Based on the GC content (Figure 5), the *dnd*-encoding GIs displayed mosaic structures and can be separated into several modules. Further examination on the presence of ORFs from the *dnd*-encoding GIs in other *E. coli* genomes further supported their mosaic characteristics. The *dnd* modification systems, *dpt* restriction systems, together with the hypothetical proteins in between the two systems were collectively found associated with *dnd*-positive genomes only (ORFs depicted by green- coloured bars). On the other hand, majority of the ORFs outside the *dpt*- and *dnd*-clusters are present in both *dnd*-positive and *dnd*-negative *E. coli* genomes (brown-coloured bars, Figure 5), with the exception of an integrase gene (*E. coli* B7A) and 6 ORFs at the immediate upstream of *dnd* operon in KOEGE118, which are found associated with *dnd*-positive genomes only. Intriguingly, some hypothetical proteins are strain-specific, which can be observed in *E. coli* strain 55989 (yellow-coloured bar).

### Different genetic contents were identified at the *leu* tRNA insertion sites of *dnd*-negative and *dnd*-positive *E. coli* genomes

All *dnd*-encoding GIs determined in our study were associated with tRNA, an integration hotspot in bacterial genomes [18]. This prompted us to investigate the genetic contents at the same tRNA gene site (*leu* tRNA) in *dnd*-negative *E. coli* genome. The tRNA insertion sites of four genomic regions (1 *dnd*-negative and 3 *dnd*-positive) from *E. coli* strains CFT073, UMEA 3176-1 , B7A and 55989 were compared (Figure 6). Upstream flanking regions of the genomic islands which were inserted into the *leu* tRNA gene sites for all 4 *E. coli* genomes are highly conserved, indicating similar upstream backbone (Figure 6). While a 15.6 kb non-*dnd*-encoding GI (consists mainly of hypothetical proteins) was found attached to the *leu* tRNA gene site of *E. coli* CFT073, the similar attachment sites in *E. coli* strains B7A, UMEA 3176-1 and 55989 were occupied with *dnd*-encoding island of variable sizes (53.3 kb – 59.5 kb). The downstream flanking regions of genomic islands for *dnd*-negative (*E. coli* CFT073) and *dnd*-positive (*E. coli* UMEA 3176-1 and B7A) genomes are also conserved (Figure 6), indicating that the *leu* tRNA gene site in *E. coli* is a variable region with other types of GIs found in *dnd*-negative strains. Despite the highly conserved upstream and downstream flanking regions for *dnd*-encoding GIs for *E. coli* strain UMEA 3176-1 and B7A, slight variations within the highly similar GIs were still observed with transposases found in the variable regions (Figure 6).

## Discussion

PFGE is often regarded as the “gold standard” for subtyping *E. coli*. However, Dnd^+^*E. coli* strains that were untypeable from our previous studies yielded incomplete molecular epidemiological data. The addition of thiourea to the PFGE running buffer has enabled us to overcome the untypeable problem of all 12 Dnd^+^*E. coli* strains. In our study, 9/12 Dnd^+^ strains were represented by two distinctly different pulsotypes [S1-S7 (n = 7); A1-A2 (n = 2)] (Figure 1), suggesting that the Dnd^+^ phenotype might be an inherent clonal trait for strains with the two respective pulsotypes. No Dnd^−^ strains were found to share similar pulsotypes with the Dnd^+^ strains when we compared the pulsotypes with previous studies [13,14]. The 9 Dnd^+^ strains were multidrug resistant with 2 extended-spectrum beta-lactamase (ESBL) producers and 7/9 were pathogenic VTEC and were clonal [13,14]. The resistance and pathogenic state of these Dnd^+^ strains may therefore pose possible threats to public health, hence improvement in their typeability is important to generate valuable epidemiological data. The pathogenic state of the Dnd^+^ strains also prompted us to further explore the strain background of all 52 *E. coli* genomes that carried *dnd* operons. Surprisingly, majority of them (n = 44/52; 85%) were pathogenic in nature with 26 being diarrheagenic *E. coli* and 18 extraintestinal pathogenic *E. coli*. To the best of our knowledge, no study has reported this finding before and the link between virulence and *dnd* operons has yet to be explored. Undoubtedly, bias might be present as pathogenic *E. coli* strains have higher level of public interest, and hence the availability of their genome data might be more compared to non-pathogenic *E. coli*. Nevertheless, this finding may provide a caveat for future studies on the possible interesting relationship between pathogenicity and the presence of the *dnd* operon in *E. coli*.

The *dnd* clusters of different bacterial species have reportedly a diverse nature [10], not only with regard to their respective sequence similarities, but also the genetic context of the *dnd* clusters as well. Some bacterial species harboured a 4-gene *dndBCDE* operon whereas others carried a five-gene *dnd* cluster (*dndA* and *dndBCDE* operon). In fact, *dndA* is not found in the *dnd* clusters of all *Enterobacteriaceae* reported in dndDB (including *E. coli*), indicating that the gene contents in the *dnd* clusters for closely related bacteria are similar. This is also the reason why *dndA* gene was not included as a target gene for prediction of degradation phenotype of *E. coli* in our study. Although DndA might be absent in some *dnd* clusters, studies have revealed that IscA can serve as functional homologues of DndA and may be found in more than one copy in the genome [15,19]. As previously reported [10], the *dnd* cluster of *E. coli* belonged to the 4-gene *dndBCDE* operon. All 12 Dnd^+^*E. coli* strains in this study were positive for the *dndB*-*dndE* genes and the presence of *dndB*-*dndE* genes was significantly associated with the DNA degradation phenotypes (p < 0.5). This indicates that the presence of these genes can be a potential genetic marker to predict the phenotype of DNA degradation. A previous study [12] developed an assay to predict the DNA degradation phenotype of *Mycobacterium abscessus* based on the fact that the *dnd* gene cluster was associated with a genomic island of the *M. abscessus* complex and the genetic environment of the *dnd*- encoding GI was highly conserved. One pair of primers targeted the *dndC* gene while another pair confirmed the absence of the *dnd* operon. If the *dnd* operon is present, the amplicon will be too large to be amplified. On the other hand, if the *dnd*-encoding GI is absent, a short bridge amplicon spanning the GI insertion site will be generated for Dnd^−^*M. abscessus* strains [12]. However, this approach was not applicable in our study due to the variable genetic environment of the *E. coli dnd* operons based on MSA analysis. Hence for *E. coli*, the detection of all 4 *dndB*-*dndE* genes would be a better alternative to confirm the presence of the *dnd* operon.

The dissemination of *dnd* operons was reportedly facilitated by mobile genetic elements especially GIs [10,19]. Hence, representative *E. coli* genomes harbouring *dnd* operons were subjected to genomic island determination. Our data showed that all the *dnd* operons in the selected genomes were in fact located in GIs. Out of 31 *dnd* operons described in dndDB, 29 were also reported to be located in chromosomal islands, one was plasmid-encoded while another one is located in a plasmid-derived chromosomal segment. These results showed the potential role of genomic islands in facilitating the horizontal gene transfer of *dnd* operons. Genomic islands have also been known to be the mechanism of diversification contributing to bacterial evolution [20]. However, it should be noted that majority of the genomes of *E. coli* in this study were draft genomes. Thus, we were unable to ascertain if the *dnd*-encoding GIs identified were chromosomally located or plasmid-encoded. Further analysis would be necessary to verify their location.

The genetic environment of the *dnd* operon in *E. coli* is very diverse (Additional file 3: Figure S2 and Figures 3, 4, 5 and 6). On the other hand, the *dnd* operon together along with its immediate vicinity in *Erwinia amylovora* and *Salmonella enterica* appeared to be conserved within the same bacterial species (Figure 2 and Additional file 3: Figure S2), indicating that their genetic environment could be similar. Analysis of representative *E. coli* genomes has shown that their respective *dnd* operons were associated with genomic islands that possess diverse genetic context. Different genetic fragments of the island that corresponded to regions of different types of *E. coli* genomes (*dnd*-positive or *dnd*-negative) indicate that the genetic elements were likely acquired from different origins. Genetic variation can also be observed among highly conserved *dnd*-encoding GIs due to insertion or deletion of transposases along with other hypothetical proteins (Figure 6). A large cluster of T6SS was also found inserted into the upstream of another *dnd*-encoding island (Figure 4). Furthermore, marked difference in GC content was also observed across the *dnd*-encoding GIs, supporting the mosaic composition of the GIs carrying *dnd* operons. The mosaic structure of the *dnd*-encoding GIs (Figure 5), presence of phage-like integrases and presence of transposable elements flanking the GIs as well as across the *dnd*-encoding GIs (Figure 6), suggest multiple integration or recombination events, which has also been reported by He et al., (2007) [19] for the *dnd*-encoding GI of *Streptomyces lividans* 66. Insertion and deletion of genetic elements as well as recombination appear to play an essential role in the constant evolution of these GIs.

Besides the diverse genetic context of the *dnd*-encoding GIs of *E. coli*, the tRNA genes associated with the islands can also be variable. The 3′-end of tRNA genes frequently served as the insertion sites for foreign DNA fragments [18]. All 9 *dnd*-encoding GIs described in this study were associated with tRNA genes, but only limited to three types (*leu*, *thr* and *phe* tRNA genes) (Figures 3 and 4), which have also been reported to be among the most frequently targeted tRNA genes for insertion in *E. coli* [18,21]. One of the possible reasons that particular tRNA genes often served as an integration hotspot is that the integrases of GIs may have specificity for certain tRNA genes [22]. For *E. coli* KOEGE 118 and KTE 9 which harboured two *dnd*-encoding GIs within the same genome, their respective *dnd*-encoding GIs were also associated with different tRNA genes (*leu* and *thr* tRNA genes) (Figure 4). However, it should be noted that not all GIs are associated with tRNA genes [23].

This is the first report describing two *dnd* operons found in the same bacterial genome. However, the benefits to its host, if any, are unknown. Several advantages to bacteria with DNA phosphorothioation modification have been proposed. One of them is that the modified host DNA is able to act as an antioxidant as phosphorothioation gave the modified DNA chemical reduction property. Thus, the modified DNA is resistant to growth inhibition and oxidative double-stranded DNA breakage caused by H_2_O_2_ [9]. H_2_O_2_ is generated during aerobic metabolism and studies have revealed that aerobic *E. coli* can generate high levels of H_2_O_2_ that are toxic enough to cause damage to their own DNA. On the other hand, exogenous H_2_O_2_ can also be found in the environment as competing organisms can release H_2_O_2_ as toxin to suppress the growth of competitors [24]. Bacteria can get killed by high levels of H_2_O_2_ generated endogenously and exogenously. Hence it is essential to protect the host bacteria against damage caused by peroxide and DNA that has been modified by phosphorothioation was hypothesized as one of the mechanisms that can offer protection to the host [24]. Nevertheless, the biological implication of having two copies of the *dnd* operons within a genome is currently unknown.

All the *dnd*-encoding GIs identified in this study also harboured *dptFGH*, a set of three conserved *dnd*-linked orthologs which are often found near the *dnd* operon. *E. coli* KTE9 and KOEGE118 which harboured 2 *dnd*-encoding GIs in the same genome also carried the three conserved genes within the islands. The conserved *dnd*-linked orthologs have been proven to function as restriction systems in *Salmonella enterica* serovar Cerro87 [25] and recently, in *E. coli* B7A [26]. The restriction system is able to restrict foreign DNA (such as phages and plasmids) that lacks the specific phosphorothioation modification when the host DNA is protected by the modification, and hence prevents the invasion of heterologous DNA [25]. Phosphorothioate DNA modifications incorporated by Dnd proteins (DndABCDE) that work together with the DptFGH- restriction system function as a restriction-modification (R-M) system, which is evident in *E. coli* B7A [26]. However, the same study also ruled out other known R-M mechanisms, namely the types I, II, III and IV R-M systems [26], implying that the Dpt-Dnd system is likely a new R-M-type mechanism. These two systems which are located in the same genomic island provide selective advantages to the bacterial host and hence may be stably maintained [12,27]. Nevertheless, the three conserved *dnd*-linked orthologs were not identified in all *dnd* encoding GIs described in previous studies [10,19] but only in several diverse bacterial strains such as *E. coli*, *Hahelia chejuensis*, *Oceanobacter* sp., and *Bacillus cereus*. This serves to underline not only the diversity of the genetic environment surrounding the various *dnd* operons but also their possible roles in their respective hosts. Perhaps they are like toxin-antitoxin systems which have multiple biological functions depending on their location in the genome and their respective hosts [28] which have adopted and adapted these genetic modules for cellular function. Intriguingly, previously described *dnd*-encoding GI of *Geobacter uraniireducens* Rf4 was also found to carry putative toxin-antitoxin loci [29]. The presence of the *dnd* operons in such varied genomic islands in *E. coli* and their biological implication will hopefully be revealed in the near future.

## Conclusion

The designed PCR assay and the use of thiourea in PFGE may improve the detection and typeability of Dnd^+^*E. coli* strains. Our study showed that genomic islands not only play a potential pivotal role in facilitating the horizontal gene transfer of *dnd* operons, but also essential in generating diversity within the genomic islands of closely related bacteria. Different *dnd*-enoding genomic islands with mosaic compositions were identified within *E. coli*. Since the *dnd* operon is often associated with mobile genetic elements, the operon may be integrated into genetically diverse strains followed by dissemination. Therefore the Dnd^+^ phenotype can be observed in both genetically closely related and diverse strains. The finding that *dnd* operons were more often found in pathogenic *E. coli* may provide caveat for future study on their possible linkage.

## Methods

### Pulsed-field gel electrophoresis

A total of 12 *E. coli* strains that were reported to be untypeable from previous studies [13,14] were further analysed in this study. Seven of the Dnd^+^ strains were verotoxigenic- *E. coli* (VTEC) isolated from different piglets in the same swine farm. The other five were clinical samples obtained from two medical centers (A and B) and from different sources (stool, blood, urine, and swab). The DNA degradation observed in these strains was thought to be Tris-dependent due to the characteristic smearing pattern and their untypeability was confirmed by repeating the PFGE three times. PFGE was carried out on *Xba*I-digested and *Bln*-digested genomic DNA of both Dnd^+^ and Dnd^−^ strains [13] with and without the addition of 50 μM thiourea (Sigma Aldrich, USA) into the 0.5 × TBE buffer and agarose gels [3]. *Xba*I-digested *Salmonella enterica* serovar Braenderup H9812 was used as the DNA size marker.

### PCR assay detecting *dndB*, *dndC*, *dndD* and *dndE* genes

A PCR assay targeting the internal sequences of the *dndB*, *dndC*, *dndD* and *dndE* genes were developed based on the *dnd* operons of 3 Dnd^+^*E. coli* genome sequences (B7A, 55989 and SE11 with accession numbers of CP005998.1, NC_011748 and NC_011415, respectively) using Primer3 (http://www.ncbi.nlm.nih.gov/tools/primer-blast/) [30]. PCR detection of *iscS* gene was also carried out using primers described previously [11]. PCR detection of *dndB*, *dndC*, *dndD*, *dndE* and *iscS* genes was carried out for 12 Dnd^+^ (which displayed the degradation phenotype) and 48 Dnd^−^ (without degradation phenotype) strains. Five each of *Shigella sonnei*, *Salmonella* serovar Typhimurium and *Salmonella* serovar Enteriditis strains that do not display DNA degradation phenotype were also included as negative controls. The presence of all 4 *dnd* genes was indicative of the presence of the *dnd* operon. Strain that did not yield any of the expected amplicons was considered as negative for Dnd genotypes. PCR amplified *dndB*, *dndC*, *dndD*, and *dndE* genes were sequenced at a commercial facility (First BASE Laboratories).

### Association of the DNA degradation phenotypes and genotypes

Fisher’s exact test was used to determine the association of the DNA degradation phenotypes and genotypes using R (version 2.12.2, Vienna, Austria, [www.R-project.org]) [31]. A significance level of p < 0.05 was considered as statistically significant.

### Comparative analysis of *dnd* operons with 1kb of their respective immediate vicinity

The three complete *dnd* operons of Dnd^+^*E. coli* strains (B7A, 55989 and SE11) available from dndDB (*dnd* database, http://db-mml.sjtu.edu.cn/dndDB) were used as query sequences because the *dnd* operons as well as their association with genomic islands (GI) have been reported [10]. BLASTN against GenBank databases of nr/nt and WGS was carried out with *Enterobacteriaceae* set as ‘organism of selection’ [32]. The sequences of *dnd* operons of all positive hits were then extracted.

The immediate vicinity of the *dnd* operon was also included by using a uniform distance of 1000 bp upstream and downstream from the *dnd* operon. A distance of 1000 bp was selected because PCR can then be employed to examine the immediate upstream and downstream of the *dnd* operon of the studied strains. Furthermore, the 1000 bp region should be sufficient to determine the neighboring gene(s) of the *dnd* operon since an ORF of *E. coli* generally has an average length of approximately 1000 bp [33]. All positive hits were considered as *dnd* operon positive although the Dnd phenotypes for most hits have not been reported before. Genetic relationships of the *dnd* operons and together with their immediate genetic environment were determined by constructing phylogenetic trees using maximum-likelihood (ML) method with MEGA 5. The *dnd* operons of *E. coli* genomes were further sub-grouped based on their respective immediate upstream and downstream regions as different genetic environments may indicate different evolutionary origins.

### Identification, designation and analysis of *dnd*-encoding genomic islands in *E. coli*

One *E. coli* genome was selected from each of the subgroups (based on their respective immediate genetic environment) for genomic island identification. In cases where only draft genomes were available in the subgroup(s), the genome was selected based on the longest assembled contig where the *dnd* operon is located. Genomic islands harbouring the *dnd* operon were determined using the VRprofile server (http://bioinfo-mml.sjtu.edu.cn/VRprofile/) and mGenomeSubtractor tool [34]. The common features of a genomic island such as organism-atypical G + C contents, integration into the 3′-end of tRNA genes, and integrase-encoding sequences were also analyzed for the putative laterally acquired *dnd*-encoding GIs. A standardized naming system for *dnd*-encoding GIs was also proposed in this study to avoid confusion. Using “dndI_I_EC 55989_” as an example, “dndI” means “*dnd*-encoding genomic island”; “_I” means “first *dnd*-encoding GI identified in the respective strain” while “EC 55989” depicts the “strain code”. The genetic maps, GC contents of representative genomic regions and *dnd*-encoding GIs were illustrated using Easyfig [35]. BLASTN comparison between the *dnd*-encoding GIs were performed with default settings using Easyfig [35]. Homologues search against the GenBank databases of nr/nt and WGS using BLASTN (http://blast.ncbi.nlm.nih.gov/blast) were carried out for ORFs of representative *dnd*-encoding genomic islands, to determine their match to *dnd*-negative or *dnd*-positive bacterial genomes.

### PCR determination of immediate genetic environment of *dnd* operons for 12 Dnd^+^*E. coli* strains

Primers targeting at the immediate upstream and downstream of the *dnd* operon were also designed based on the 52 sequences extracted from the *E. coli* genomes. A total of 13 pairs of primers were designed based on the 7 types of genetic environment found for *E. coli* (Table 2). The primers are summarized in Table 2 and were used to determine the immediate genetic environment for the 12 Dnd^+^*E. coli* studied strains.

**Table 2:** **Primers used to determine the immediate genetic environment of** ***dnd*** **operons of** ***E. coli*** **strains**

**Primer**	**Oligonucleotide sequence (5' to 3')**	**Target gene**	**Amplicon size (bp)**	**Annealing temperature (°C)**	**Reference(s)**
gp_a_US-F	AACAACTGTGGGTCAGCGAA	upstream of	489	52	this study
gp_a_US-R	AGGCTATCTGATGCTCCCGA	*dnd* operon			
gp_b_US-F	TGGGGGTTGAGTCTGATGAT	upstream of	723	52	this study
gp_b_US-R	TCGAATGGTGCTGAGTCGTC	*dnd* operon			
gp_cI_US-F	GAAACCAACCCTCTTTTCACGTC	upstream of	823	52	this study
gp_cI_US-R	GTCTGATGCTCCCGAATCGAA	*dnd* operon			
gp_cII_US-F	ACCATCGAAAGCCCCATTAAGAG	upstream of	760	52	this study
gp_cII_US-R	GCTGTCTGATGCTCCCGAAT	*dnd* operon			
gp_d_US-F	AGGCTGGAAGCCATGTTTTG	upstream of	905	52	this study
gp_d_US-R	TTCGTCGATCAACTGCGTGA	*dnd* operon			
gp_e_US-F	CCATATGTCAGCTCAAGTCGC	upstream of	612	52	this study
gp_e_US-R	TACTGATACGGAAGTGGATGAGC	*dnd* operon			
gp_f_US-F	GGGCGAGTTCGATGCTATGAC	upstream of	595	53	this study
gp_f_US-R	TGGGGGAGACACTACAAGCTA	*dnd* operon			
gp_a_DS-F	ATTCGTGCCGGGAAACTCAT	downstream of	636	52	this study
gp_a_DS-R	TCAGTGCTGTGCGTAGTGAG	*dnd* operon			
gp_b_DS-F	CGCTTTACGACGATGCTGAC	downstream of	591	52	this study
gp_b_DS-R	GCGAAACCAAGACGTGG(A/T)CT	*dnd* operon			
gp_c_DS-F	CGTTGAGCGCGTAATTCTGG	downstream of	582	52	this study
gp_c_DS-R	ATCAGGGGCTTCTTGCAGAC	*dnd* operon			
gp_d_DS-F	CGCGTCCATCGGTACACATA	downstream of	558	52	this study
gp_d_DS-R	TGCCACTTCAGTGCTGACAA	*dnd* operon			
gp_e_DS-F	AACGGCAATAGACGCTGTCA	downstream of	518	52	this study
gp_e_DS-R	AGCAACCGTCTTCGTTCTGT	*dnd* operon			
gp_f_DS-F	ACATCTGCTGGCTACGCTTT	downstream of	715	52	this study
gp_f_DS-R	TTGTCATGCGGTCTTAGCGA	*dnd* operon			

## Additional files

Additional file 1: Figure S1. Multiple sequence alignment of *dnd* genes using Clustal W. a) *dndB* gene; b) *dndC* gene; c) *dndD* gene; d) *dndE* gene. A dot (‘.’) denotes a position at which all query sequences have the exact same nucleotide sequence. Nucleotide differences in reference and query sequences are indicated as underlined and uppercased alphabets, respectively. Numbers indicate the nucleotide sequence positions. Nucleotide sequences for *E. coli* 559891, SE115 and B7A were retrieved from dndDB [GenBank: NC_011748, NC_011415, CP005998.1, respectively]. PPN8b-EC2^2^, EC1003-8^3^, EC1004-8^4^, EC062/10^6^ and EC075/10^7^ represent strains in lanes S3, A1, A3, B1 and B2 in Figure 1. Additional file 2: Table S1. Strains included in phylogenetic analysis of *dnd* operon. *E. coli* strain background information is also included. Additional file 3: Figure S2. Phylogenetic tree of *dnd* operons together with their respective immediate genetic environment (1kb) from *Enterobacteriaceae*. Asterisks (*) indicate that the genome contains two copies of *dnd* operons. Different alphabets (a, b, cI, cII, d, e, f) depict the subgroups based on the immediate genetic environment. Maximum likelihood (ML) method was used to construct the phylogenetic tree using MEGA5. Bootstrap values have been calculated using 1000 replicates. Bootstrap confidence values greater than 50% are shown at branches. Nodes with less than 50% bootstrap value are collapsed.

## References

[1] Van Belkum A, Tassios P, Dijkshoorn L, Haeggman S, Cookson B, Fry NK (2007). Guidelines for the validation and application of typing methods for use in bacterial epidemiology. Clin Microbiol Infect.

[2] Fawley WN, Wilcox MH (2002). Pulsed-field gel electrophoresis can yield DNA fingerprints of degradation-susceptible *Clostridium difficile* isolates. J Clin Microbiol.

[3] Romling U, Tummler B (2000). Achieving 100% typeability of *Pseudomonas aeruginosa* by pulsed-field gel electrophoresis. J Clin Microbiol.

[4] Silbert S, Boyken L, Hollis RJ, Pfaller MA (2003). Improving typeability of multiple bacterial species using pulsed-field gel electrophoresis and thiourea. Diagn Microbiol Infect Dis.

[5] Zhou X, Deng Z, Firmin JL, Hopwood DA, Kieser T (1998). Site-specific degradation of *Streptomyces lividans* DNA during electrophoresis in buffers contaminated with ferrous iron. Nucleic Acids Res.

[6] Zhou X, He X, Li A, Lei F, Kieser T, Deng Z (2004). *Streptomyces coelicolor* A3(2) lacks a genomic island present in the chromosome of *Streptomyces lividans* 66. Appl Environ Microbiol.

[7] Zhou X, He X, Liang J, Li A, Xu T, Kieser T (2005). A novel DNA modification by sulphur. Mol Microbiol.

[8] Ray T, Mills A, Dyson P (1995). Tris-dependent oxidative DNA strand scission during electrophoresis. Electrophoresis.

[9] Xie X, Liang J, Pu T, Xu F, Yao F, Yang Y (2012). Phosphorothioate DNA as an antioxidant in bacteria. Nucleic Acids Res.

[10] Ou HY, He X, Shao Y, Tai C, Rajakumar K, Deng Z (2009). dndDB: a database focused on phosphorothioation of the DNA backbone. PLoS One.

[11] An X, Xiong W, Yang Y, Li F, Zhou X, Wang Z (2012). A novel target of IscS in *Escherichia coli*: Participating in DNA Phosphorothioation. PLoS One.

[12] Howard ST, Newman KL, McNulty S, Brown-Elliott BA, Vasireddy R, Bridge L (2013). Insertion site and distribution of a genomic island conferring DNA phosphorothioation in the Mycobacterium abscessus complex. Microbiology.

[13] Ho WS, Tan LK, Ooi PT, Yeo CC, Thong KL (2013). Prevalence and characterization of verotoxigenic-*Escherichia coli* isolates from pigs in Malaysia. BMC Vet Res.

[14] Ho WS, Balan G, Puthucheary S, Kong BH, Lim KT, Tan LK (2012). Prevalence and characterization of multidrug-resistant and extended-spectrum beta-lactamase-producing *Escherichia coli* from pediatric wards of a Malaysian hospital. Microb Drug Resist.

[15] Wang L, Chen S, Vergin KL, Giovannoni SJ, Chan SW, Demott MS (2011). DNA phosphorothioation is widespread and quantized in bacterial genomes. Proc Natl Acad Sci U S A.

[16] Fontecave M, Ollagnier-de-Choudens S (2008). Iron-sulfur cluster biosynthesis in bacteria: Mechanisms of cluster assembly and transfer. Arch Biochem Biophys.

[17] Bellanger X, Payot S, Leblond-Bourget N, Guédon G: Conjugative and mobilizable genomic islands in bacteria: evolution and diversity. FEMS Microbiol Rev 2014, doi: 10.1111/1574-6976.12058. 10.1111/1574-6976.12058 24372381

[18] Ou HY, Chen LL, Lonnen J, Chaudhuri RR, Thani AB, Smith R (2006). A novel strategy for identification of genomic islands by comparative analysis of the contents and contexts of tRNA sites in closely related bacteria. Nucleic Acids Res.

[19] He X, Ou HY, Yu Q, Zhou X, Wu J, Liang J (2007). Analysis of a genomic island housing genes for DNA S-modification system in *Streptomyces lividans* 66 and its counterparts in other distantly related bacteria. Mol Microbiol.

[20] Juhas M, van der Meer JR, Gaillard M, Harding RM, Hood DW, Crook DW (2009). Genomic islands: tools of bacterial horizontal gene transfer and evolution. FEMS Microbiol Rev.

[21] Germon P, Roche D, Melo S, Mignon-Grasteau S, Dobrindt U, Hacker J (2007). tDNA locus polymorphism and ecto-chromosomal DNA insertion hot-spots are related to the phylogenetic group of *Escherichia coli* strains. Microbiology.

[22] Boyd EF, Almagro-Moreno S, Parent MA (2009). Genomic islands are dynamic, ancient integrative elements in bacterial evolution. Trends Microbiol.

[23] Langille MGI, Hsiao WWL, Brinkman FSL (2010). Detecting genomic islands using bioinformatics approaches. Nature Rev Microbiol.

[24] Park S, You X, Imlay JA (2005). Substantial DNA damage from submicromolar intracellular hydrogen peroxide detected in Hpx-mutants of *Escherichia coli*. Proc Natl Acad Sci U S A.

[25] Xu T, Yao F, Zhou X, Deng Z, You D (2010). A novel host-specific restriction system associated with DNA backbone S-modification in *Salmonella*. Nucleic Acids Res.

[26] Cao B, Chen C, DeMott MS, Cheng Q, Clark TA, Xiong X (2014). Genomic mapping of phosphorothioates reveals partial modification of short consensus sequences. Nat Commun.

[27] Wang L, Chen S, Deng Z: Phosphorothioation: An Unusual Post-Replicative Modification on the DNA Backbone. In DNA Replication-Current Advances. Edited by Dr Herve S. ISBN: 978- 953-307-593-8, InTech, Available from: http://www.intechopen.com/books/dna-replication-current-advances/phosphorothioation-an-unusual-post-replicative-modification-on-the-dna-backbone.

[28] Chan WT, Moreno-Córdoba I, Yeo CC, Espinosa M (2012). Toxin-antitoxin genes of the Gram-positive pathogen *Streptococcus pneumoniae*: so few and yet so many. Microbiol Mol Biol Rev.

[29] Shao Y, Harrison EM, Bi D, Tai C, He X, Ou HY (2010). TADB: a web-based resource for Type 2 toxin-antitoxin loci in bacteria and archaea. Nucleic Acids Res.

[30] Ye J, Coulouris G, Zaretskaya I, Cutcutache I, Rozen S, Madden T (2012). Primer-BLAST: A tool to design target-specific primers for polymerase chain reaction. BMC Bioinformatics.

[31] R Development Core Team: R: a language and environment for statistical computing. Available at www.R-project.org. Vienna, Austria: R Foundation for Statistical Computing, 2008. ISBN 3-900051-07-0. accessed May 2014.

[32] Altschul SF, Gish W, Miller W, Myers EW, Lipman DJ (1990). Basic local alignment search tool. J Mol Biol.

[33] Brown TA: Chapter 7, Understanding a Genome Sequence. In Genome. 2nd edition. Oxford: Wiley-Liss; Available from: http://www.ncbi.nlm.nih.gov/books/NBK21136/

[34] Shao Y, He X, Tai C, Ou HY, Rajakumar K, Deng Z (2010). mGenomeSubtractor: a web-based tool for parallel in silico subtractive hybridization analysis of multiple bacterial genomes. Nucleic Acids Res.

[35] Sullivan MJ, Petty NK, Beatson SA: Easyfig: a genome comparison visualiser. Bioinformatics 2011, doi:10.1093/bioinformatics/btr039. 10.1093/bioinformatics/btr039 PMC3065679 21278367

