# Supplementary material for: The dnd operon for DNA phosphorothioation modification system in Escherichia coli is located in diverse genomic islands
Source: BMC Genomics. 2015 Mar 17;16(1):199. doi: 10.1186/s12864-015-1421-8 (PMC4373003; doi:10.1186/s12864-015-1421-8)
Supplement: Additional file 1: Figure S1. — Multiple sequence alignment of dnd genes using Clustal W. a) dndB gene; b) dndC gene; c) dndD gene; d) dndE gene. A dot (‘.’) denotes a position at which all query sequences have the exact same nucleotide sequence. Nucleotide differences in reference and query sequences are indicated as underlined and uppercased alphabets, respectively. Numbers indicate the nucleotide sequence positions. Nucleotide sequences for E. coli 559891, SE115 and B7A were retrieved from dndDB [GenBank: NC_011748, NC_011415, CP005998.1, respectively]. PPN8b-EC22, EC1003-83, EC1004-84, EC062/106 and EC075/107 represent strains in lanes S3, A1, A3, B1 and B2 in Figure 1. [file 12864_2015_1421_MOESM1_ESM.pdf]

a) *dndB*

```
             1                                                                                                 99
55989[1]     CGTAAAGCGATTGAAGACGCTTTAGACGCTCGCCCGGAGCTGGGGCAGGATAACATACCTGTGCTGTTTTTTGTCGATGAAGGATTAAAACGCAGCCAA
PPN8b-EC2[2] ...................................................................................................
EC1003-8[3]  ...................................................................................................
EC1004-8[4]  ...................................................................................................
SE11[5]      ......................................A.................T..........................................
EC062/10[6]  ..........................T............T................T..G.....................G.................G
EC075/10[7]  ................................T...........C........T..C..G...T....C..............C.....G..T.....G
B7A[8]       ................................T...........C........T..C..G...T....C..............C.....G..T.....G
             100                                                                                               198
55989        CAGATGTTTGCCGATCTGAATAAATATGCTATTCGCCCCAGTCCGTCGTTGGCATCGTTGTATGATCATCGGGATGCCAGTTCAAATTTGGCGCGTTAT
PPN8b-EC2    ...................................................................................................
EC1003-8     ...................................................................................................
EC1004-8     ...................................................................................................
SE11         ...................................T.....C..A..........................................C...........
EC062/10     ........C..T.....A........C..C..............T..AC........C....C........T..C..T........CC...........
EC075/10     ........C.................C..C...........C..A...C....G...C.A..C........C......T....G...C...........
B7A          ........C.................C..C...........C..A...C....G...C.A..C........C......T....G...C...........
                     207
55989        CTGGCAATG
PPN8b-EC2    .........
EC1003-8     .........
EC1004-8     .........
SE11         .........
EC062/10     .........
EC075/10     .........
B7A          .........
```

b) *dndC*

```
             1                                                                                                 99
55989        CCCCGGAAATGCGCAACAAAGATGTTTTTGTAGTGTCATCCGATACGCTGGTGGAAACACCAGTGGTAGTAGATTTAATTAAGAAAACGATGTTGCAAA
PPN8b-EC2    ...................................................................................................
EC1003-8     ...................................................................................................
EC1004-8     ...................................................................................................
SE11         ...............G..........................................C.....A................................G.
EC062/10     .............T.GT..............T.....G..............A........G..A..G..G...C.C...........C........G.
EC075/10     ...............G.......A.......G.....G..............C........G..A..C..G...C.G....................G.
B7A          ...............G.......A.......G.....G..............C........G..A..C..G...C.G....................G.
             100                                                                                               198
55989        TTGAGGCAGGCGCAAAGCGTAATGGCCTGCCGATCACTCAACATGCGGTAACGCCTAAAACTAATGAAACCTTCTGGGTCAATTTACTGGGGAAAGGTT
PPN8b-EC2    ...................................................................................................
EC1003-8     ...................................................................................................
EC1004-8     ...................................................................................................
SE11         ................A..................................................................................
EC062/10     .C..A..C.....T..A.........T....A..T....................G.....C...........T.....T.....G.....T.....C.
EC075/10     .......G..A..G........C...T................C..................G.......................G.............
B7A          .......G..A..G........C...T................C..................G.......................G.............
             199                                                                                               297
55989        ATCCGGCCCCAACCCGCAGTTTTCGCTGGTGTACCGAACGCATGAAAATCAATCCGGTGAGTGACTTTATTAAAGAAAAGGTCAGCCAGTTTGATGAAG
PPN8b-EC2    ...................................................................................................
EC1003-8     ...................................................................................................
EC1004-8     ...................................................................................................
SE11         ................T....................G..T....................C..................A..................
EC062/10     ....T.....T.....T....................G..T...........C.....T..C..............T..A...........C.......
EC075/10     .......T.....T..T.....C..T.....C..T..G..............C.......................T......................
B7A          .......T.....T..T.....C..T.....C..T..G..............C.......................T......................
             298                                                                                               396
55989        TAATTGTTGTCCTAGGTTCACGTAGTAGTGAGAGTGCCTCTCGTGCGCAGGTCATCGCAAAGCACAAAATTGATGGCTCACGCTTGGCCCGGCATACCA
PPN8b-EC2    ...................................................................................................
EC1003-8     ...................................................................................................
EC1004-8     ...................................................................................................
SE11         .G...........C..C.......................C..........................................................
EC062/10     .G.....G..GT.G..A..G..C..C..C..A.....T........T.....G........A..............G..T..AC.T...........T.
EC075/10     .C..C..C..A..T.....G..C........A.....T...............G..............G........A......C.T.............
B7A          .C..C..C..A..T.....G..C........A.....T...............G..............G........A......C.T.............
             397                                                                                               495
55989        CACTGGCAAATGCGTTTATCTATACCCCTATTGATACCTGGGATGTAGAAGACGTCTGGAAACTGCTGCGCGGCGCGTTCCGCTATGCTCCTGAATATA
PPN8b-EC2    ...................................................................................................
EC1003-8     ...................................................................................................
EC1004-8     ...................................................................................................
SE11         .G.............................................................................................G...
EC062/10     .C.....G.....C.....T.....G..C.......G.........C.....T........GT.............A.....A............G...
EC075/10     .G...........A..C...........C...........................T........G........G.................C......G...
B7A          .G...........A..C...........C...........................T........G........G.................C......G...
                                     520
55989        TCGACGAATGGGAAAGCCCGTGGGG
PPN8b-EC2    .........................
EC1003-8     .........................
EC1004-8     .........................
SE11         .........................
EC062/10     .T..T....................
EC075/10     .......G.................
B7A          .......G.................
```

c) *dndD*

```
            1                                                                                                  99
55989       TGTGTCCGTCAGGTGCAAAACTGCATGATGCGGCCCGCAGTCAGCATGGCTTTAGTAGCGCCTTCAAAAATGCGCAGGAAACAATTAATTTACTCGAT
PPN8b-EC2   .....T.............................................................................................
EC1003-8    .....T.............................................................................................
EC1004-8    .....T.............................................................................................
SE11        .....T........A..G...GCA.....CATCA....T..C..A.....T..G..C.....A..T.................C.....CC.G......
EC062/10    .....T...........................................................T....................C............
EC075/10    .....T......A.T......GC......C..C..T..TTA......CA.T.A...C..T..G..T..G.....T........G..A............
B7A         .....T......A.T......GC......C..C..T..TTA......CA.T.A...C..T..G..T..G.....T........G..A............
            100                                                                                                198
55989       CGGTATAGCGACGTATTAACTCAGGCGAGAGTAAAGACGCTATCTGCTAACTTTGAAGTTGCCTATCGCAAACTGGCTCGTAAAGAAGATATGCAACTG
PPN8b-EC2   ...................................................................................................
EC1003-8    ...................................................................................................
EC1004-8    ...................................................................................................
SE11        TAC..C.....T...C.T..G......C....G..A.AAT....G............A.......C.A..................G...T.A..G..T
EC062/10    ...................................................................................................
EC075/10    ..C..C.....T........C.....TC.G.....A.....G..GA.A.....C..GC....T..C..T..GT.......C.....G...T.A.....T
B7A         ..C..C.....T........C.....TC.G.....A.....G..GA.A.....C..GC....T..C..T..GT.......C.....G...T.A.....T
            199                                                                                                297
55989       AGTGCTCATATTAACCCAGAGACATTTGACGTTGAGTTAATTGATGAGAAGGGATCTGTCATTAATCGTAAGTTACTATCTGCCGGTGAAAAGCAGATT
PPN8b-EC2   ...................................................................................................
EC1003-8    ...................................................................................................
EC1004-8    ...................................................................................................
SE11        .A...G.....A..T..GC..........T.....AC.GG.G.....A.....T..G..G............C.G..T.....G...............
EC062/10    .............................T........................G.................C......................A...
EC075/10    .....G.....C.....TC.A..G.....T.....A..............T..C..A..A.....C...........T.....T........A......
B7A         .....G.....C.....TC.A..G.....T.....A..............T..C..A..A.....C...........T.....T........A......
            298                                                                                                396
55989       TATGCCATTGCTATTCTTGAGGCGCTAGCAAAAACTTCTGGTCGTGATTTGCCGGTGATTATTGATACGCCATTAGGACGTCTGGATTCACAACATCGG
PPN8b-EC2   ...................................................................................................
EC1003-8    ...................................................................................................
EC1004-8    ...................................................................................................
SE11        .....G.....C..............G........C.....G........C....................G.....G...........T..G......
EC062/10    ..........................G........................................................................
EC075/10    .....A....................G..G.....C.....C........A..A.....C........A.....G..T........C..T........A
B7A         .....A....................G..G.....C.....C........A..A.....C........A.....G..T........C..T........A
            397                                                                                                495
55989       GATAAACTAATTAACCATTATTTCCCATTTGCCAGCCATCAAGTGGTGCTATTGTCTACCGATACGGAAGTGGATGAACGTTATTTTGTTGACCAATTA
PPN8b-EC2   ...................................................................................................
EC1003-8    ...................................................................................................
EC1004-8    ...................................................................................................
SE11        ......T.G.....T...........GGAA...........G..T.....GC.A..C........T........C..G........C..C..T..C..G
EC062/10    ...........................................................T.......................................
EC075/10    ......T.G.....T..C..C.....TGAA.....T.....G..T...T.GC.T...........T.....A....................T..G..G
B7A         ......T.G.....T..C..C.....TGAA.....T.....G..T...T.GC.T...........T.....A....................T..G..G
            496                                                   551
55989       CGTGATGATATTTCTCATGCTTACGAGATAGTATTTAACACGCATACTAAATCATC
PPN8b-EC2   ........................................................
EC1003-8    ........................................................
EC1004-8    ........................................................
SE11        .......................T.....T..G.....TG................
EC062/10    ..C.....................................................
EC075/10    ...........C...........T.....T..G.....TG.A........G.....
B7A         ...........C...........T.....T..G.....TG.A........G.....
```

d) *dndE*

```
            1                                                                                                  99
55989       TCGGTGGAGAGTGAGTTTCGCTATTCATCAGAACGGGATAATCGGAAACTAGATGGTTCGCTGGTGTTGGATAAGATTACGTGGCTGGGGGAGACATTA
PPN8b-EC2   ...................................................................................................
EC1003-8    ...................................................................................................
EC1004-8    ...................................................................................................
SE11        ..........................GC.T..G.......G.AA...G..G.....C.....A...C.......A...........T.....A...C.G
EC062/10    ...........................C.G....................G.......................A........................
EC075/10    ...........................C.T.....A....G.AA...G..G......A........C.......A.....................C..
B7A         ...........................C.T.....A....G.AA...G..G......A........C.......A.....................C..
            100                                                                                            194
55989       CAGACTACGGAATTGGTATTGAAGATGCTTTATCCGCAGTTAGCTCAGAAGGATATGGTTAAAGCATGGGCTGCGCATGTGGAGGATGGGATTGC
PPN8b-EC2   ................................................................................T........A..C..
EC1003-8    ................................................................................T........A..C..
EC1004-8    ................................................................................T........A..C..
SE11        ..AG....A...C....G..A..............TA.TC.C.AC..A..ATTG..AA.............A..A.....T........A..C..
EC062/10    ............C...................................................................T........A..C..
EC075/10    ..AG....A........GC................A..A....AG..A....CGT..A....G...........T.....T........A..C..
B7A         ..AG....A........GC................A..A....AG..A....CGT..A....G...........T.....T..............
```

Figure S1: Multiple-sequence alignment of *dnd* genes using Clustal W.
