# Supplementary material for: The dnd operon for DNA phosphorothioation modification system in Escherichia coli is located in diverse genomic islands
Source: BMC Genomics. 2015 Mar 17;16(1):199. doi: 10.1186/s12864-015-1421-8 (PMC4373003; doi:10.1186/s12864-015-1421-8)
Supplement: Additional file 3: Figure S2. — Phylogenetic tree of dnd operons together with their respective immediate genetic environment (1kb) from Enterobacteriaceae. Asterisks (*) indicate that the genome contains two copies of dnd operons. Different alphabets (a, b, cI, cII, d, e, f) depict the subgroups based on the immediate genetic environment. Maximum likelihood (ML) method was used to construct the phylogenetic tree using MEGA5. Bootstrap values have been calculated using 1000 replicates. Bootstrap confidence values greater than 50% are shown at branches. Nodes with less than 50% bootstrap value are collapsed. [file 12864_2015_1421_MOESM3_ESM.pdf]

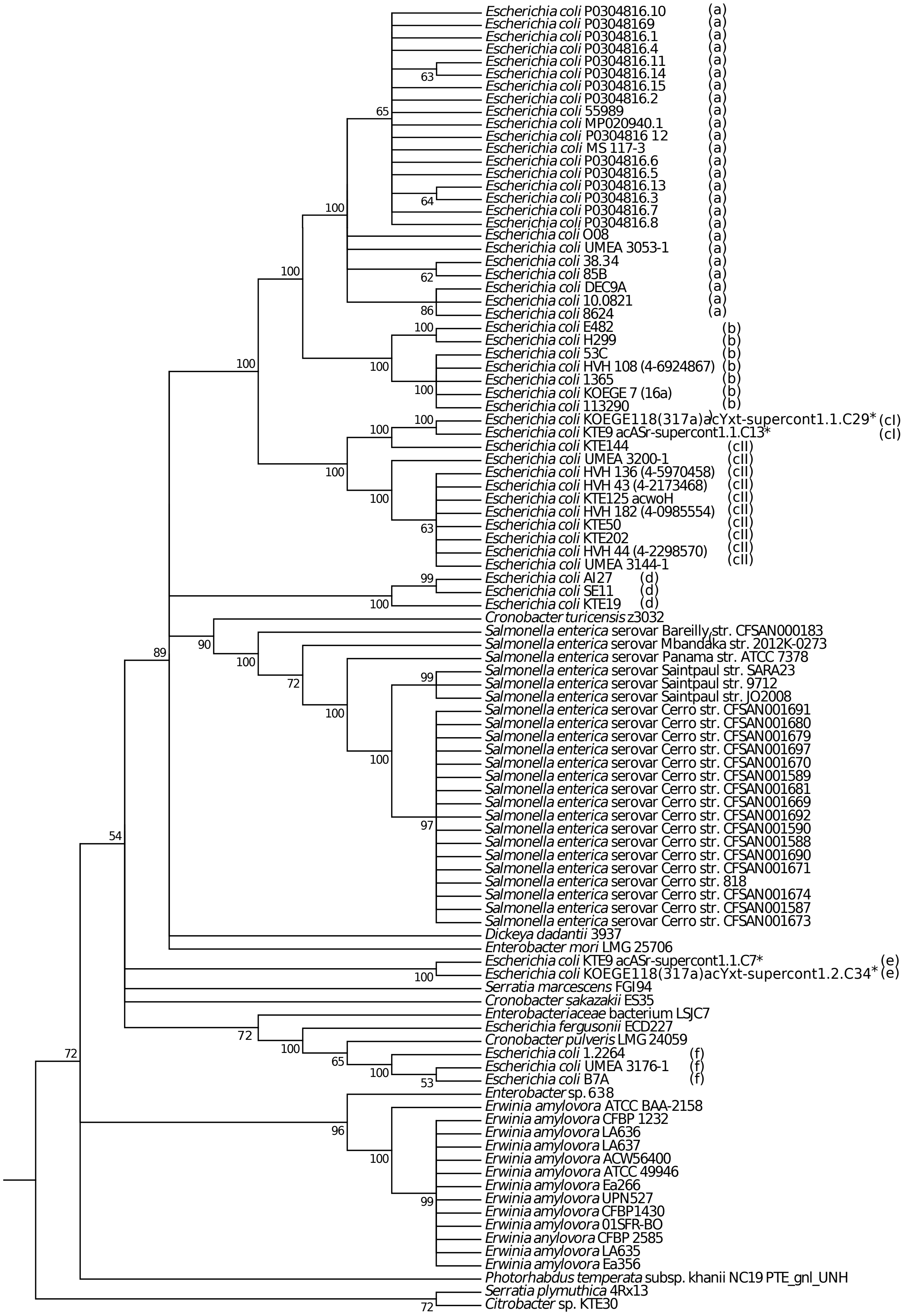

Figure S2. Phylogenetic tree of *dnd* operons together with their respective immediate genetic environement (1kb) from *Enterobacteriaceae*.
